# Artificial mixed microbial system for polycyclic aromatic hydrocarbons degradation

**DOI:** 10.3389/fmicb.2023.1207196

**Published:** 2023-06-15

**Authors:** Jia-Qi Cui, Zhi-Qiang He, Samuel Ntakirutimana, Zhi-Hua Liu, Bing-Zhi Li, Ying-Jin Yuan

**Affiliations:** ^1^Frontiers Science Center for Synthetic Biology and Key Laboratory of Systems, Bioengineering (Ministry of Education), Tianjin, China; ^2^School of Chemical Engineering and Technology, Tianjin University, Tianjin, China

**Keywords:** PAHs, bioremediation, artificial MMS, synthetic biology, process intensification

## Abstract

Polycyclic aromatic hydrocarbons (PAHs) are environmental pollutants with major risks to human health. Biological degradation is environmentally friendly and the most appealing remediation method for a wide range of persistent pollutants. Meanwhile, due to the large microbial strain collection and multiple metabolic pathways, PAH degradation *via* an artificial mixed microbial system (MMS) has emerged and is regarded as a promising bioremediation approach. The artificial MMS construction by simplifying the community structure, clarifying the labor division, and streamlining the metabolic flux has shown tremendous efficiency. This review describes the construction principles, influencing factors, and enhancement strategies of artificial MMS for PAH degradation. In addition, we identify the challenges and future opportunities for the development of MMS toward new or upgraded high-performance applications.

## Graphical abstract

Graphical Abstract 
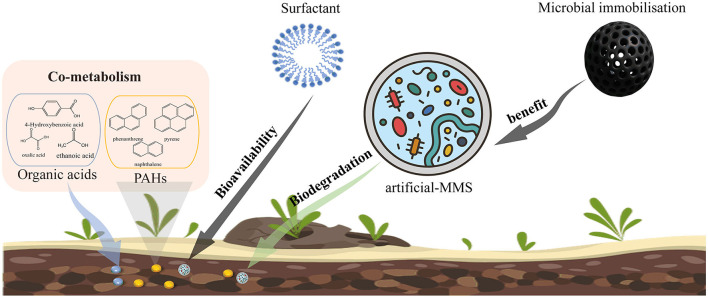

## 1. Introduction

With the rapid development of the global economy, a large number of non-renewable fossil-based fuels (coal, oil, and gas) are always needed for mining and refining, in order to provide various types of functional compounds to improve industrial processes and living quality (Cui et al., 2020). Among them, petroleum and its derivatives play a crucial role in human activities. However, the reserves of non-renewable resources are not only sharply decreased yearly but also the ecological environment is easily polluted during fossil fuel refining and transportation (Johnston et al., 2019). Petroleum is mainly composed of carbon, hydrogen, oxygen, and other elements, and its hydrocarbons could be divided into alkanes (paraffin and naphthalene), aromatic hydrocarbons (BETX-benzene, toluene, ethylbenzene and xylene, and PAHs-polycyclic aromatic hydrocarbons), and asphaltenes. In previous research, it had been found that PAHs have a negative effect (toxicity, teratogenicity, and mutagenesis) on organs and tissues (Kuppusamy et al., 2017); thus, these aromatic hydrocarbons have been regarded as priority pollutants by the US Environmental Protection Agency (EPA) (Gan et al., 2009; Imam et al., 2021). In fact, these aromatic pollutants are not only originated from the production and utilization of petroleum but also from straw burning and volcanic eruption (Gu et al., 2022).

**Figure 1:**
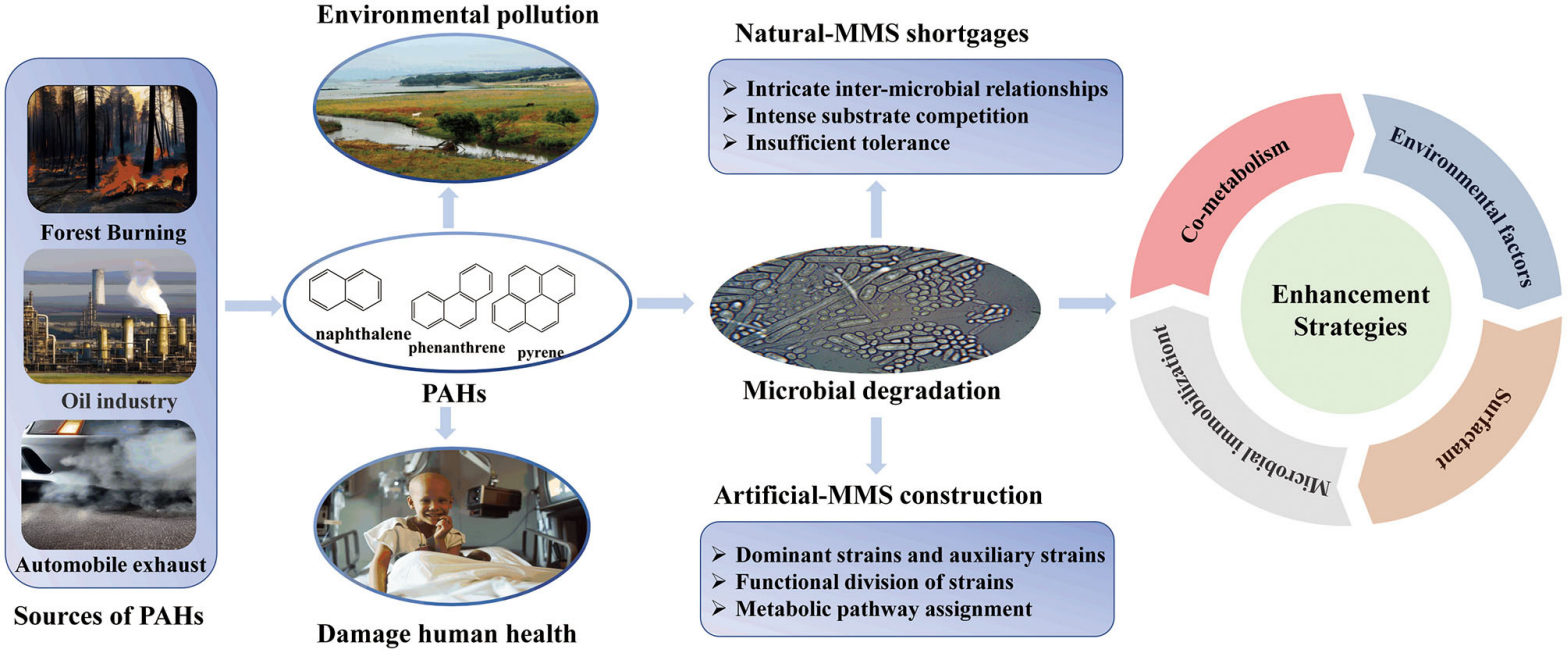
PAH sources and microbial degradation enhancement strategies.

To date, remediation technology has attracted the great attention from environmental protection agencies around the world, and various PAH degradation strategies (physical, chemical, physicochemical, and biological methods) have achieved very gratifying results (Grossman et al., 2019). Although the remediation methods thereof are faster with high efficiency, their high cost, complexity, and secondary pollution risk limit their effectiveness for meeting the needs of sustainable remediation. Owing to its wide source, multiple metabolic pathways and environmentally friendly, biological methods have been extensively applied to remediate the PAHs-contaminated sites (Figure 1) (Mehetre et al., 2019; Varjani et al., 2020). However, PAH biodegraders sourced from petroleum hydrocarbon (PH)-polluted sites have shown limited adaptation with a low degradation rate. Hence, it is an important element to further explore and amplify the effectiveness of biodegraders' potential for PAHs removal.

In the bioremediation process of PAHs, bacteria, fungi, and archaea are the major biodegraders to remove hydrocarbons at the polluted site, and the specific-gene (*alk, nah*, and *pahE*) and metabolic pathways (fatty acids break down, tricarboxylic acid cycle) were used under anaerobic and aerobic conditions to degrade and transform PAHs into harmless substances (such as carbon dioxide and water) (Ghosal et al., 2016; Duarte et al., 2017; Liang et al., 2019; Dou et al., 2021). Owing to the wide PAHs metabolism pathways, stronger environmental adaptability, and faster reproduction ability, bacteria have been the prevalent biodegraders. Recently, various species of bacteria, such as *Pseudomonas, Bacillus, Mycobacterium, Rhodococcus*, and other alpha-, beta-, and gamma-proteobacteria, have been studied extensively for PAH degradation (Duarte et al., 2017; Srivastava and Kumar, 2019). Furthermore, mycoremediation has also gained popularity due to its co-metabolic activity. Unlike bacteria, fungi do not utilize PAHs as sources of carbon and energy, but they act like an engineered system for allocating nutrients and energy through their mycelial network (El Amrani et al., 2015). So far, bio-augmentation (single-microorganism or mixed-microorganism inoculation) and bio-stimulation (nutrients addition) are the main strategies to improve the microorganism (indigenous) activity and mineral contents in the contaminated area for bioremediation (Xu and Zhou, 2016).

During the bioremediation of PAHs, single-bacteria or mixed bacteria have been utilized, where the bacteria are regarded as key microorganisms for hydrocarbon degradation (Khan et al., 2021; Varjani and Upasani, 2021). When single-bacteria with higher PAHs degradation ability were added to pollution sites, the carbohydrate biodegradation and microorganism growth were enhanced in the early stage of remediation. Unfortunately, the biodegradation activity of inoculated single bacteria gradually decreases with the extension of the bioremediation period, thus attenuating the degradation efficiency of PAHs in the polluted sites. Compared to single-bacteria, mixed-bacteria exhibited a higher bacteria diversity, abundant carbon metabolism pathway, and stronger stress resistance, and it was widely applied to polluted sites (Patel et al., 2019). Although the mixed-bacteria exhibit improved PAHs bioremediation efficiency, it still has some limitations, such as redundant flora structure, complex metabolic pathways, and fierce substrate competition (Wu et al., 2022). Therefore, further enhancement of bioremediation efficiency of mixed-bacteria through simplified microorganism structure clarified labor division, and carded metabolic flux will become the key nodes for increasing bioremediation potential for PAH pollutants. This review focuses on the construction principles, influence factors, and optimization strategies of artificial mixed microbial system (MMS). Finally, it is concluded with the perspectives on the future development of this bioremediation technology.

## 2. Artificial MMS construction strategy

**Figure 2:**
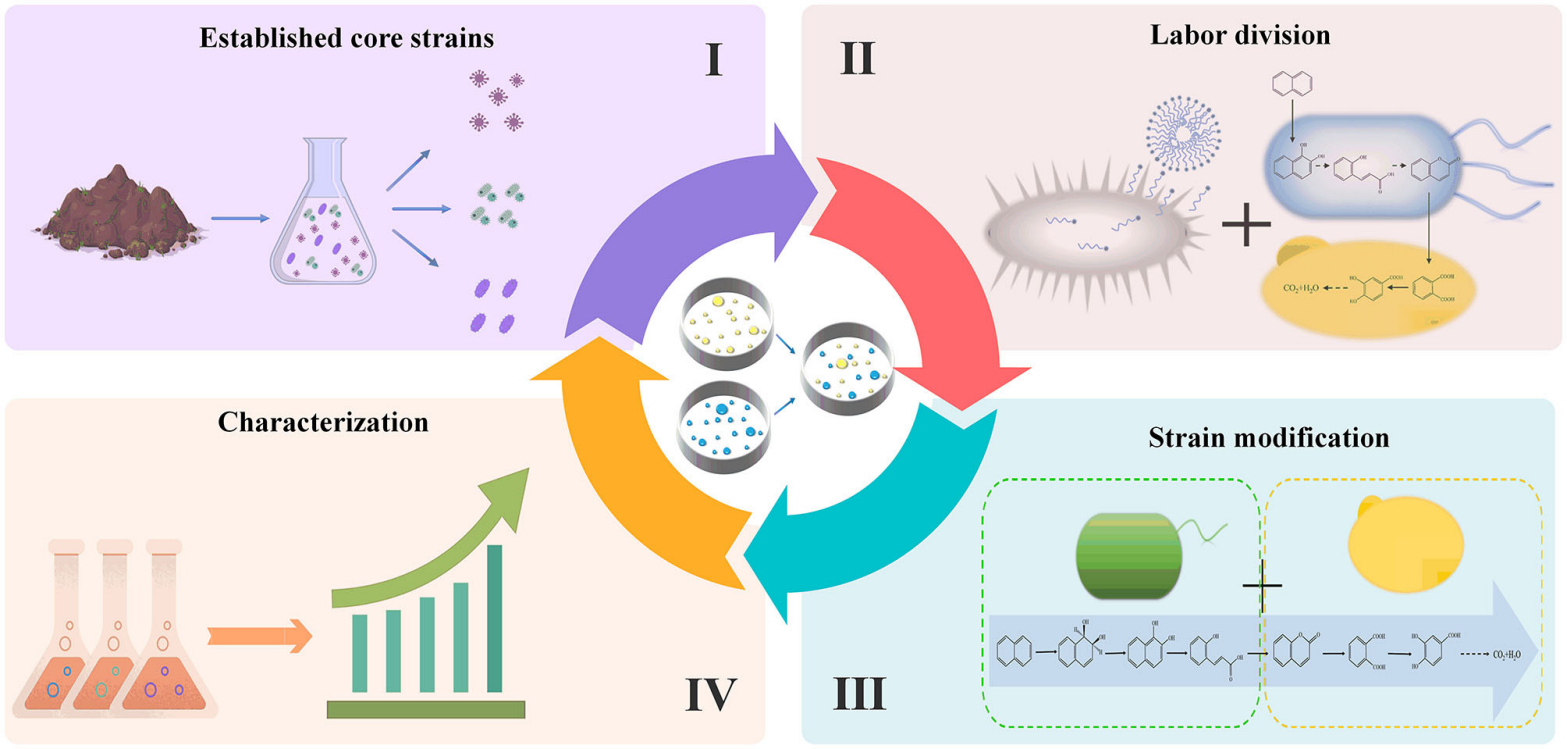
Artificial MMS construction and influence factors.

In nature, most PAH-degrading bacteria lack mature and effective genetic modification methods, whereas the artificial MMS construction provides a new direction for effective PAH removal. At present, MMS has shown positive outcomes in environmental pollutant remediation and solid waste management, and the dominant strains and critical gene modules in these systems have been identified through gene sequencing and system biology techniques (Premnath et al., 2021; Wang et al., 2022) (Table 1). However, traditional MMS has presented challenges, such as high microbial diversity, complex metabolic networks, and intense substrate competition. Therefore, based on previous studies and experimental results, this section presents construction strategies of artificial MMS and eventually gets the goals of quickly, accurately, and efficiently enhancing the degradation efficiency of PAHs by the bioremediation method (Figure 2).

**Table 1:** MMS for PAH degradation properties characterization.

**Main strains**	**Pollution**	**Intermediate**	**Critical Gene**	**Efficiency**	**Reference**
*Azoarcus Chelativorans*	PHE FLT	Catechol Phthalic acid Salicylic acid	—	—	Patel et al. (2019)
*Pseudomonas Sphingobium*	PHE DBT NAP FLU DBF	—	PAH-PHDα *pahE*	100% (mixed PAHs) time <5 d	Zhang et al. (2021d)
*Pseudomonas aeruginosa* DAK11.1 *Pseudomonas stutzeri* DAK11.2 *Achromobacter sp*. DAK11.3 *Chelatococcus sp*. DAK11.4	NAP PHE FLT PYR	Oct 9-OCT Tet 9,10-Eth etc.	—	27% (NAP) 51% (PHE) 16% (FLT) 19% (PYR) time <5 d	Patel et al. (2018)
*P. monteilii* P26 *Pseudomonas* sp. N3 *Gordonia* sp. H19 *Rhodococcus* sp. F27	NAP PHE PYR	—	—	90% (NAP) 80% (PHE) 70% (PYR) time <8d	Isaac et al. (2015)
*Pseudomonas* sp. *Rhodococcus* sp.	PYR	—	*catABC nahCF pcaGH benA-xylX benB-xylY benC-xylX benD-xylL* etc.	41.69% (PYR) time <5 d	Su et al. (2022)
*Methylobacterium Burkholderia Stenotrophomonas*	PHE PYR	—	—	94% (PHE) 96.2% (PYR) time <7 d	Li et al. (2021b)
*Rhodococcus* sp. WB9 *Mycobacterium* sp. WY10	PHE	1-hydroxy-2-naphthoic acid diphenic acid phthalate protocatechuate	—	93% (PHE) time <36 h	Sun et al. (2021)
*Pseudomonas aeruginosa Alcaligenes faecalis*	NAP PHE PYR	—	—	90% (NAP) 80% (PHE) 70% (PYR) time <8 d	Zhang et al. (2022)
*Pseudoxanthomonas Dokdonella Starkeya Hydrogenophaga*	PHE	—	—	95.41% (PHE) time <48 h	Jiang et al. (2022)
*Massilia* sp. WF1 *Phanerochaete chrysosporium*	PHE	—	PAH-RHDα	95.92% (PHE) time <35 d	Gu et al. (2021)

NAP, naphthalene; PHE, phenanthrene; FLU, fluorene; FLT, fluoranthene; PYR, pyrene; DBT, dibenzothiophene; DBF, dibenzofuran; Oct, Octane, 3, 4, 5, 6-tetramethyl; 9-OCT, 9-octadecenoic acid (Z), phenyl methyl ester; Tet, tetrahydropyran-2-yl ester; 9,10-Eth, 9, 10-Ethanoanthracene, 9, 10-dihydro-11, 12-diacetyl; PAH-PHDα, PAH hydrotase aldolase genes; *pahE*, PAH ring-hydroxylating dioxygenase genes; *catABC*(*catA, catB, catC*), catechol 1,2-dioxygenase gene, muconate cycloisomerase gene, muconolactone d-isomerase gene; *nahC*, 1,2-dihydroxynaphthalene dioxygenase gene; *nahF*, salicylaldehyde dehydrogenase; *pcaGH*(*pcaG, pcaH*), protocatechuate 3,4-dioxygenase; *benA-xylX, benB-xylY*, benzoate 1,2-dioxygenase gene; *benC-xylZ*, ring-hydroxylating dioxygenase gene; *benD-xylL*, 1,6-dihydroxycyclohexa-2,4-diene-1-carboxylate dehydrogenase gene.

### 2.1. Established core strains

A large number of microorganisms with strong tolerance and degradation ability for aromatic compounds have been discovered and used for environmental remediation. Among them, PAH degradation by bacteria is a major research trend, and the removal efficiency has been significantly improved by MMS (Moscoso et al., 2012; Gosai et al., 2022; Patel et al., 2022). However, neither single-bacteria nor MMS (active sludge and composting systems) met expectations for PAH degradation efficiency, thus simplifying composition and establishing the core strain of MMS is a crucial method for addressing the aforementioned challenges.

Among PAH biodegraders, *Bacillus* spp. and *Acinetobacter* have been reported to possess a diverse array of hydrocarbon metabolic types, pathways, and genes. For example, *Bacillus* sp. PAH-2 and *Bacillus sphaericus* have been shown to degrade both PYR and BaP simultaneously (Hunter et al., 2005; Kong et al., 2022). Meanwhile*, Acinetobacter* has also been shown to possess the ability to degrade a variety of PAHs, such as PHE, PYR, and anthracene (Jiang et al., 2018). However, these bacteria have some limitations, such as uncompleted carbon metabolic pathways and the production of toxic metabolites (Flowers-Geary et al., 1996). To enhance the PAH degradation performance of artificial MMS, the inoculation ratio and time of dominant bacteria and auxiliary bacteria should be optimized, then the bioconversion efficiency of hydrocarbon by the above MMS could be improved.

### 2.2. Artificial MMS labor division

The traditional MMS with high microbial diversity and widely metabolic network needs to be enhanced for PAH biodegradation levels (Haritash, 2020). However, the conversion potential of aromatic compounds among microorganisms was also limited by metabolism substrate and pathway interference. Therefore, the bacteria with different functionalities were selected to construct artificial MMS, and the degradation efficiency of PAHs could increase by simplifying the structure of MMS.

It was reported that *Cupriavidus* sp. PH2 and *Escherichia coli* HY1 can establish an artificial MMS for PHE removal. The *Cupriavidus* sp. PH2 was a PHE-degrading strain, and the expression level of catechol 1,2-dioxygenase was enhanced after introducing the dioxygenase gene (from *Alcanivorax borkumensis* SK2) and the *nidA*+*nidB* gene (from *Mycobacterium*) into *E. coli* HY1. Compared to the wild type (*Cupriavidus metallidurans*), PHE oxidation and ring cleavage were performed by *E. coli* HY1 and *Cupriavidus* sp. PH2, respectively, thus the degradation rates of PHE were significantly increased (Jia et al., 2019). Meanwhile, when three modules (aromatic ring cleavage module, salicylic acid synthesis module and PHE metabolism module) were introduced separately into *E. coli* BL21, which produced engineered strains M1, M2 and M3, respectively, then the coculture of the above engineered strains as an artificial consortium for the biodegradation of the contents, it resulted in decreased PAHs from 100 mg/L to 27.33 mg/L within 7 days (Zhang et al., 2021a).

The biodegradation of PAHs was improved by simplifying the structure of microorganisms, but there is still space for enhancing the utilization of hydrocarbon with rational strategies. Due to PAHs with low bioavailability, a surfactant-enhanced bioremediation method was developed, and the interface tension of oil–water was effectively reduced, and ultimately improved the restoration potential of artificial MMS for the contaminated site (Martinez-Toledo et al., 2022). During biodegradation by a two-species microbial consortium, a biosurfactant-secreting bacteria (*Pseudomonas aeruginosa*) was introduced into artificial MMS, and the biodegradation rates of PHE were significantly increased from 61.15% to 73.86% (Qin et al., 2022). The strong ability of cell proliferation and PAH degradation was shown by single bacteria (Llasera et al., 2021), but the above strain usually lacks a comprehensive metabolic pathway and enzyme system for hydrocarbons. Therefore, PAHs bio-degrader was regarded as a core strain and combined with multi-function bacteria was a targeted and high-potential bioremediation strategy for hydrocarbon metabolism, then a low-cost, targeted, and high-potential bioremediation strategy was constructed.

### 2.3. Enhancement of key metabolic pathways

This study aimed further to improve the PAH conversion of artificial MMS, the function of bacteria could be re-designed and modified to enhance inter-cooperation efficiency. Owing to the lack of full genes and enzymes/enzyme systems of PAH conversion in bacteria, the open-loop genes (such as the *phn* gene clusters and *nah*-like gene clusters) and hydrocarbon degradation pathways could be introduced into core strain by molecular engineering strategies. Some studies have indicated that the biodegradation of PHE and PYR in soil could be enhanced by over-expression of the *nahH* gene (encoding catechol 2,3-dioxygenase) in *Stenotrophomonas maltophilia* (Mardani et al., 2017). During the exogenous gene *C23O* that encoded catechol 2,3-dioxygenase was introduced into the alkane-degrading bacterium *Acinetobacter sp*. BS3, the recombinant strain BS3-*C23O* showed a significant increase in its ability to degrade a wide range of PAHs and n-alkanes (Xie et al., 2014). Meanwhile, the directed evolution of P450-BM3 in *Escherichia coli* resulted in improved activity and coupling efficiency of the engineered variant toward the non-native substrate acenaphthene (ACN) (Maxel et al., 2020). It was also reported that the PAH bioconversion genes and pathways of core or subordinate strain were rationally modified and arranged by molecular biology methods, thereby improving the hydrocarbon metabolic flow and enzyme secretion capacity in artificial MMS for PAH removal (Pant et al., 2021).

## 3. Influence factors of artificial MMS

While the PAH degradation by the microbial method has an outstanding efficiency in hydrocarbon conversion, the bio-removal level is affected by microorganism activity and environmental factors. In addition to the above factors, the remediation of contaminated sites by artificial MMS is also limited by inter-bacterial relationships, colony stability, and substrate distribution. This section focuses on the factors that impact the PAH metabolism in artificial MMS.

### 3.1. Multiple hydrocarbon pollutants

The situation of PH contaminated site is complex, which PAHs co-existing with other contaminants (alkanes, cycloalkanes, and heavy metals), and multi-functional MMS is crucial for PAHs bioremediation. For a suitable microbial strategy, it is necessary to assess the types and concentrations of hydrocarbons, mineral element contents (carbon, nitrogen, and phosphorus), and environmental factors (salinity, water content, and conductivity) at contaminated sites (Varjani, 2017). In the multi-substrate condition, it is an important factor to screen and construct an appropriate MMS for various pollutants' degradation, meanwhile, bio-consortium with multiple-metabolic patterns is also major (Zhang et al., 2021d). A multi-functional artificial MMS was built with respect to the pollutants, which enhanced the remediation efficiency of the microbial system. It is common that contaminated sites are polluted by heavy metals and PAHs, while the presence of heavy metal ions can damage the functional enzymes of PAH-degrading bacteria (Ali et al., 2022). Therefore, an engineered MMS containing both PAH-degrading and heavy metal ion-removing bacteria can effectively remediate complex contamination sites.

### 3.2. Inter-bacterial adaptability and stability

For traditional MMS, the relative abundance of strain and substrate-dependent competition of dominant bacteria was higher than that of non-dominant bacteria, meanwhile, the growth of non-dominant bacteria was maintained by intermediate metabolites. In contrast, a rational simplified and optimized artificial MMS displayed enhanced PAH degradation and bioremediation potential, but the degradation efficiency of different functional microorganisms was inhibited by high substrate competition and poor compatibility (Mille-Lindblom et al., 2006).

Although a large number of microorganisms with PAH degradation ability have been isolated and identified, 99% of potential hydrocarbon degraders are yet to be discovered in nature, and the adaptability of artificial MMS would be further improved by expanding the scope of strain discovery. Currently, molecular sequencing technology and bioinformatics are regarded as useful tools to explore the relationship of inter-bacterial interaction, such as mutualism and competition, and its impact on the compatibility within the artificial MMS (Yu et al., 2022).

It is crucial to effectively maintain the viability of MMS microorganisms at contaminated sites (wastewater or soil). As it is well known, a single strain or artificial MMS directly introduced into a new environment would result in an obvious decrease of exogenous microorganism richness or even quickly disappeared in the pollution sites.

The addition of supplemental nutrients to contaminated sites can enhance the activity of microbial strains, and it will provide a buffer for exogenous MMS to adapt to the local environment. Moreover, the combined biostimulation with the bioaugmentation process is more effective than bioaugmentation alone in increasing microbial community diversity, maintaining structural balance, and improving remediation efficiency.

### 3.3. Pathways modification

Recently, the artificial MMS has exhibited high efficiency toward hydrocarbon conversion, but there is still space for further improvement in the remediation potential for PAH pollutants. The existence of high microbial diversity and complex metabolic networks in traditional MMS results in high substrate competition and metabolic interference among microbes.

With the advancement of molecular biology and bioinformatics, intracellular metabolic fluxes or key targets are simulated and predicted to modify microbial genetic information and have become a powerful approach to explore suitable transformation methods of complex substrates by microorganisms (Jaiswal and Shukla, 2020; Xiang et al., 2021). The accurate construction of macrogenome metabolic network model can be used to design a simplified artificial MMS and predict the key genes and limiting rate targets of PAH metabolism (Gosai et al., 2022). Additionally, the weighted co-expression network analysis (WGCNA) was utilized to visualize and identify critical genes or metabolic pathways of PAH biodegradation, which aimed to enhance the accuracy of strain modification. Concurrently, several gene editing technologies have been developed, such as CRISPR/Cas9 and its derivative gene editing tools (*S. pyogenes* Cas9-derived base editor, Prime Editor), which provide robust support for precisely modifying and reconstructing core genes and metabolic pathways in the artificial MMS (Komor et al., 2017; Doman et al., 2020; Li et al., 2020; Rafeeq et al., 2023).

### 3.4. Environmental factors

The PAH removal efficiency of artificial MMS is not only affected by the aromatic hydrocarbon metabolism of strain but also environmental factors have a significant impact on the bioremediation of contaminated sites. The mineral ratio of carbon (C), nitrogen (N), and phosphorus (P) (C: N: P = 100: 10: 1) has displayed a dominant role in the biodegradation of PAHs during microbial metabolism, while an increase in carbon concentrations led to an imbalance in the ratio of nutrients at contaminated sites (water or soil), thus the aromatic hydrocarbon conversion potential of artificial MMS should be enhanced by nutrients' exogenous addition.

The biodegradation ability of indigenous microorganisms was improved through the biostimulation of marine sediments by supplementation of digestate (Bianco et al., 2020). Recently, aerobic pathways were primarily utilized for PAH biodegradation by most microorganisms (Baboshin and Golovleva, 2012; Elyamine et al., 2021; Liang et al., 2023), and oxygen has served as a suitable electron donor for microbial growth and metabolism, so it is crucial to optimize the contents of oxygen to accelerate the conversion of PAHs. Meanwhile, due to the low biodegradability of aromatic hydrocarbon, the solubility of PAHs was increased by adding surfactants (Bolan et al., 2023). In addition, moisture content, salinity, and electron transfer agents play an important role in the microbial strategy for solid–waste remediation (Li et al., 2021c).

## 4. Process optimization of PAHs biodegradation

**Figure 3:**
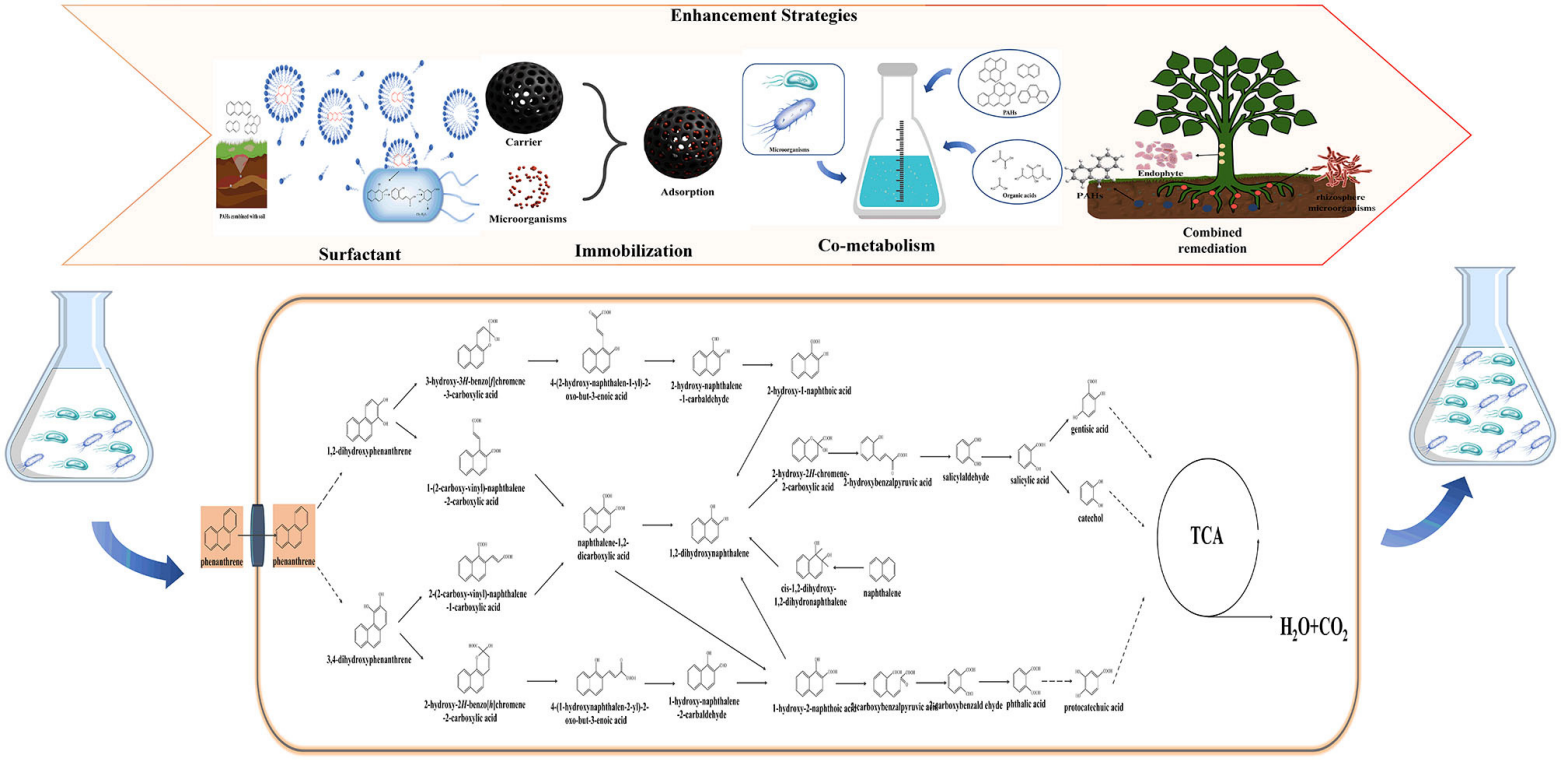
Promotion strategies for artificial MMS of PAH degradation.

It was an important point that knowing both PAHs physico–chemical properties and artificial MMS characteristics, meanwhile above information could provide a guide to enhance the hydrocarbon bioavailability and microbial activity for further improving the bioremediation potential the PAHs. Therefore, this section will focus on methods that have the potential to enhance the degradation ability of artificial MMS toward PAHs, to accelerate the rate of environmental remediation through microbial strategies (Figure 3).

### 4.1. PAHs bioavailability enhancement

The aromatic hydrocarbons (particularly PAHs) are not bioavailable to microorganisms, and their absorptivity decreases with increasing molecular weight, thus significantly declining the bioavailability of PAHs. Surfactants with hydrophilic heads and hydrophobic tails were applied to the solubilization and exploitation of petroleum. Basically, surfactants are mainly divided into chemical and biological surfactants (Table 2).

**Table 2:** Enhancement strategies for PAH biodegradation.

**Strategies**	**Main strains**	**Addition**	**Pollutions**	**Control**	**Treatment**	**Reference**
Surfactant	*Kocuria rosea*	Tween-80	PHE ANT PYR	58.1% (PHE) 19.9% (ANT) 13.8% (PYR) time <7 d	70.9% (PHE) 32.5% (ANT) 54.7% (PYR) time <7 d	Khandelwal et al. (2021)
	*Pseudomonas* sp. Ph6	Rhamnolipid	PHE	64.6% (PHE) time <14 d	95.5% (PHE) time <14 days	Ma et al. (2018)
	*Pseudomonas aeruginosa* BT-1	Tween-80	BaP	61.34% (BaP) time <30 d	79.70% (BaP) time <30 d	Meng et al. (2019)
Immobilization	*Serratia* sp. AC-11	Chitosan	FLT	3.7% (FLT) time <1 d	56% (FLT) time <1 d	Garcia et al. (2019)
	*Sphingomonas* sp. GY2B	Kaolinite	PHE	85% (PHE) time <2 d	98% (PHE) time <2 d	Gong et al. (2018)
	*Klebsiella* sp.	Polyvinyl alcohol-sodium alginate-nano alumina	PHE FLT PYR	73.23% (PHE) 64.18% (FLT) 59.25% (PYR) time <30 d	89.14% (PHE) 81.25% (FLT) 78.33% (PYR) time <30 d	Xu et al. (2019)
	—	Biochar	BaP	23.5% (BaP) time <30 d	88.6% (BaP) time <30 d	Guo et al. (2021)
Co-metabolism	*B. subtilis* ZL09–26	Citric acid	PHE	17.24% (PHE)	64.99% (PHE) time <3 d	Zhang et al. (2021b)
	*Acidobacteria Alphaproteobacteria Gammaproteobacteria*	Lignin	BaA		5.55 ± 0.28% (BaA) time <56 d	Gu et al. (2022)
	—	Vanillate	BaA	4.96% (BaA) time <70 d	8.73(BaA) time <70 d	Sun et al. (2020)
	*Pseudomonas aeruginosa* PA06 *Achromobacter* sp. AC15	Sodium citrate	PYR	47.5 (PYR) time <14 d	74.6% (PYR) time <14 d	Li et al. (2021a)

PHE, phenanthrene; FLT, fluoranthene; PYR, pyrene; ANT, anthracene; BaA, benz[a]anthracene; BaP, benzo[a]pyrene.

Chemical surfactants (such as tween-80, Triton X-100, and PEG) have been widely utilized in the degradation of PAHs. It was reported that the biodegradation efficiency of MMC (*K. rosea* + *A. sydowii*) was improved by tween-80 addition, where tween-80 was preferable to be utilized by microorganism with increasing concentrations, while the carbohydrate bioconversion level was decreasing (Khandelwal et al., 2021). However, the degradation activity of bacteria could be inhibited by the toxicity of chemical surfactants. For example, the lag phase of *Moraxella osloensis* CFP312 was prolonged from 12 h to 33 h when Triton X-100 was added to the MSM medium (Xiao et al., 2021).

Compared with chemical surfactants, biological surfactants such as rhamnolipid, lipopeptide, and lichenin have shown low toxicity and environmentally friendly. Recently, rhamnolipid (below 100 mg/L) has been widely used to enhance the bioavailability of PAHs; the cell surface zeta-potential, hydrophobicity of *Pseudomonas*, and the improvement of PHE biodegradation rate were attributed to the change of ultrastructure and functional groups of cells. Moreover, with increasing concentrations of rhamnolipid (beyond 100 mg/L), the zeta-potential and hydrophobicity of cell surface decrease, which lead to retardation of absorption and degradation ability of *Pseudomonas* for PHE (Ma et al., 2018).

As the second largest biomass in nature, lignin is a type of aromatic polymer mainly present in the cell walls of plants. The presence of hydrophilic and lipophilic groups in lignin makes it a potential precursor for lignin-based surfactants (Alwadani and Fatehi, 2018). Although several modified technologies could be utilized to prepare lignin-based surfactants with excellent performance, the production cost is relatively high. Generally, direct usage of lignin obtained from biomass pretreatment as a surfactant has shown poor solubility in water, and it was not enough to decrease the surface tension of oil–liquid. Nevertheless, the lignin isolated with ethylenediamine (EDA) has shown a high solubility in water, due to the nitrogen atom introduced into the lignin structure during biomass pretreatment. Meanwhile, compared with alkali-lignin or organic-lignin, it was observed that the surface tension of oil–liquid was more reduced in the presence of EDA-lignin. Additionally, the degradation efficiency of artificial MMS toward PAHs was improved by the addition of EDA-lignin. In short, it is inferred that EDA-lignin could be an excellent lignin-based surfactant for PAH biodegradation, and it can provide a new direction for high-value application of lignin.

### 4.2. Artificial MMS activity improvement

During the bioremediation of PAH polluted areas, it is very important to maintain the bioactivity and carbohydrate metabolism of microorganisms. Meanwhile, the efficiency of PAH biodegradation is limited by biotic or abiotic factors, such as competition pressure (substrates, niche) and nutrients (nitrogen, phosphorus). Recently, microbial immobilization technology (MIT) (Table 2), which promotes oxygen transfer, maintains microbial activity, and fixes nutrients, has been widely utilized to remediate PAH-polluted groundwater or soil (Omoni et al., 2022). It was reported that carrier is a key element for MIT, where it directly impacts the activity and function of microorganisms (Gong et al., 2022).

The carriers are divided into inorganic carriers (minerals, glass, metal nanoparticles, etc.) and organic carriers (polymers, biochar, etc.), and the metal nanoparticles (size <100 nm) are the late model multifunction carriers. Due to the unique physical and chemical properties imparted by their nanoscale size, metal nanoparticles have received substantial attention in the field of pollution remediation, particularly for the removal of PAHs (Mazarji et al., 2021). It should be noted that many metal nanoparticles possess photocatalytic properties and exhibit a strong redox ability, which can have negative impacts on microorganisms. Moreover, the preparation cost of metal nanoparticles is higher compared to other carriers, which can restrict their large-scale application in environmental remediation.

Chitosan is a naturally occurring polysaccharide that is abundant in nature, and it is characterized by a high content of amino and hydroxyl functional groups, which play a positive role in binding with microorganisms. It was reported that the biodegradation of FLU was 56% within 24 h when *Serratia* sp. AC-11 was embedded in chitosan compared to only 28% of bacteria in its dissociated form (Garcia et al., 2019). Although natural polymers have poor stability and are easily decomposed, these limitations could be overcome by using synthetic polymers. Compared to inorganic carriers, biochar has a high surface area and void ratio, which allows it to both adsorb pollutants from the environment and provide a suitable habitat for microbial growth (Kong et al., 2018). As such, it is considered an ideal immobilized carrier for soil and wastewater bioremediation. When 2% of biochar (which was prepared by rape straw) was added into the soil contaminated with PAHs, the composition and diversity of bacteria in the rhizosphere of ryegrass changed, meanwhile, the activity of microorganisms and the expression levels of the RHDα GN genes increased, thus the biodegradation rates of carbohydrate were also further improved (Zhao et al., 2022).

Although biochar had displayed a great valuable impact in bioremediation, its potential risks should not be ignored. A variety of organic substances, particularly PAHs, can be formed during the production of biochar, and some researchers have reported that the average release of PAH content from 79 biochar preparation processes is 9 ± 29 mg/kg (median 0.9 mg/kg) (Buss et al., 2022). In addition, the condensation and deposition of biochar after pyrolysis can be a potential source of PAH pollution. Therefore, the content of PAHs can be reduced through an appropriate design of the pyrolysis unit, thus clean and safe biochar can provide a strong guarantee for the biological remediation strategy for PAH pollution (Buss et al., 2022).

### 4.3. Co-metabolism of simple substrates and PAHs

High-molecular weight PAHs (benzene rings beyond 3) exhibit low bioavailability and poor bioaccessibility, and they are difficult to be used as a carbon source and degraded by microorganisms. In previous studies, the PAHs' biodegradation efficiency was increased by co-metabolized substrate (glucose) in petroleum hydrocarbon-polluted groundwater or soil, while the metabolic activity of microorganisms may be inhibited for some PAHs (BaA) by the aforementioned substrate. Thus, it is a key point to explore and study more suitable co-metabolic substrates in the field of bioremediation.

At present, organic acids (citric acid, oxalic acid, and malic acid), low-molecular weight PAHs (NAP, PHE, and FLU), and lignin are regarded as potential co-metabolic substrates for PAH biodegradation (Table 2) (Zhang et al., 2021c; Gu et al., 2022). In nature, the low-molecular weight organic acids are secreted by plant roots, which provide an easy carbon source for rhizosphere microorganisms and improve their number and activity, thereby leading to the beneficial effects on PAH bioremediation (Sivaram et al., 2019). The PHE degradation efficiency of *B. subtilis* ZL09-26 was enhanced by the addition of citric acid, glutaric acid, and oxalic acid (Zhang et al., 2021b). The addition of citric acid increased the activity of bacteria, while the expression levels of cis-3,4-dihydrophenanthrene-3,4-diol dehydrogenase and phthalate 3,4-cis-dihydrodiol dehydrogenase were strengthened by the addition of glutaric acid and oxalic acid, with a subsequent increase of the PHE biodegradation.

The key factors affecting co-metabolism are the functional enzymes expressed by microorganisms and their substrate specificity. The degradation of multiple substrates is dependent on the enzymes produced by microorganisms, which are influenced by the structure of the substrates being degraded. For example, the biodegradation rates of PYR and PHE were improved by the addition of NAP, while that of NAP was decreased, which was caused by the competitive inhibition of PYR and PHE on NAP (Guha et al., 1999). Notably, the structure of lignin and its monomers are similar to that of PAHs, and they have been considered as co-metabolic substrates for the removal of petroleum hydrocarbon. Recently, the mineralization rate of BaA was enhanced after the addition of lignin and vanillin to the soil. Meanwhile, it had been verified that BaA could be degraded under the synergistic effect of fungi and bacteria, and the degradation process by fungi was related to the reaction mediated by lignin-degrading enzymes (Sun et al., 2020). In addition, the bacterial growth with the utilization of methyl and aryl groups was stimulated by the addition of lignin.

## 5. Future perspectives

In this review, an artificial MMS with approaches for improving its bioremediation efficiency with respect to PAHs at contaminated sites is discussed. Due to complex pollutants' distribution, intense substrate competition, and inter-bacterial metabolic disturbance, the artificial MMS could be further improved from the following aspects:

1. Due to the complexity and diversity of PAHs in the contaminated sites, it is difficult for natural biodegraders to meet the restoration needs of the environment, while engineered strains are considered promising biodegraders to carry out biological remediation through artificially designed and modified degradation pathways (Sharma et al., 2022). Meanwhile, the PAH degradation enhancement strategy of core strain in the artificial MMS could follow a mature process, which included selecting appropriate chassis, designing/optimizing degradation, and improving chassis tolerance (Liu et al., 2017; Xiang et al., 2021).
2. Aimed to further enhance the PAHs conversion, integrated multiple omics with macrogenome-scale metabolic network model will provide theoretical guidance for screening key metabolic pathways and rate-limiting targets in artificial MMS. In addition, new gene/cluster discovery, upgrading of data analysis tools (such as KEGG and WGCNA), and gene engineering tools (such as CRISPR, DNA assembly, and enzyme engineering) would provide a large number of supports for modifying artificial MMS (Lapinaite et al., 2020; Hathwaik et al., 2021).
3. To ensure the effectiveness of artificial MMS for remediating PAH contaminated sites, it is necessary to conduct small-scale experiments in the laboratory, to evaluate their degradation capabilities. However, these experiments are often time-consuming and may not fully simulate real wastewater or soil environments. In recent years, deep learning has made significant advances in the field of structural biology (Jumper et al., 2021), which holds great potential for predicting the construction, optimization, and remediation capabilities of artificial MMS. Finally, the design of a deep learning algorithm that leverages multifunctional performance would become an important tool for the development of a faster and more efficient bioremediation process.

## Author contributions

J-QC and Z-QH performed and prepared the manuscript. SN contributed to data collection. B-ZL and Z-HL guided the writing and editing of this manuscript. All authors contributed to the article and approved the submitted version.

## References

[B1] Ali M. Song X. Ding D. Wang Q. Zhang Z. Tang Z.. (2022). Bioremediation of PAHs and heavy metals co-contaminated soils: challenges and enhancement strategies. Environ. Pollut. 295, 118686. 10.1016/j.envpol.2021.118686 34920044

[B2] Alwadani N. Fatehi P. (2018). Synthetic and lignin-based surfactants: Challenges and opportunities. Carbon Res. Conv. 1, 126–138. 10.1016/j.crcon.07006

[B3] Baboshin M. Golovleva L. (2012). Aerobic bacterial degradation of polycyclic aromatic hydrocarbons (PAHs) and its kinetic aspects. Microbiol 81, 639–650. 10.1134/S0026261712060021 23610919

[B4] Bianco F. Race M. Papirio S. Esposito G. (2020). Removal of polycyclic aromatic hydrocarbons during anaerobic biostimulation of marine sediments. Sci. Total. Environ. 709, 6414. 10.1016./j.scitotenv.2019.136141 31887522

[B5] Bolan S. Padhye L. P. Mulligan C. N. Alonso E. R. Saint-Fort R. Jasemizad T.. (2023). Surfactant-enhanced mobilization of persistent organic pollutants: Potential for soil and sediment remediation and unintended consequences. J. Hazard. Mater. 443(Pt A), 130189. 10.1016./j.jhazmat.2022.130189 36265382

[B6] Buss W. Hilber I. Graham M. C. Masek O. (2022). Composition of PAHs in Biochar and Implications for Biochar Production. ACS. Sustain. Chem. Eng. 10, 6755–6765. 10.1021/acssuschemeng.2c00952 35634266 PMC9131514

[B7] Cui J. Liu M. He Q. Chen H. Sun M. Wen J.. (2020). Microbial community dynamics and functional responses that contribute to tolerance of high concentrations of petroleum hydrocarbon. J Chem. Technol. Biot. 95, 1361–1371. 10.1002/jctb.6320

[B8] Doman J. L. Raguram A. Newby G. A. Liu D. R. (2020). Evaluation and minimization of Cas9-independent off-target DNA editing by cytosine base editors. Nat. Biotechnol. 38, 620–628. 10.1038/s41587-020-0414-6 32042165 PMC7335424

[B9] Dou R. Sun J. Lu J. Deng F. Yang C. Lu G.. (2021). Bacterial communities and functional genes stimulated during phenanthrene degradation in soil by bio-microcapsules. Ecotoxicol. Environ. Saf. 212, 111970. 10.1016/j.ecoenv.2021.111970 33517034

[B10] Duarte M. Nielsen A. Camarinha-Silva A. Vilchez-Vargas R Bruls T. Wos-Oxley ML.. (2017). Functional soil metagenomics: elucidation of polycyclic aromatic hydrocarbon degradation potential following 12 years of in situ bioremediation. Environ. Microbiol. 19, 2992–3011. 10.1111/1462-2920.13756 28401633

[B11] El Amrani A. Dumas AS. Wick LY. Yergeau E. Berthome R. (2015). “Omics” Insights into PAH Degradation toward Improved Green Remediation Biotechnologies. Environ. Sci. Technol. 49, 11281–91. 10.1021/acs.est.5b01740 26352597

[B12] Elyamine A. M. Kan J. Meng S. Tao P. Wang H. Hu Z.. (2021). Aerobic and anaerobic bacterial and fungal degradation of pyrene: mechanism pathway including biochemical reaction and catabolic genes. Int. J. Mol. Sci. 22, 202. 10.3390./ijms22158202 34360967 PMC8347714

[B13] Flowers-Geary L. Bleczinski W. Harvey R. G. Penning T. M. (1996). Cytotoxicity and mutagenicity of polycyclic aromatic hydrocarbon o-quinones produced by dihydrodiol dehydrogenase. Chem-Biol. Interact. 99, 55–72. 10.1016/0009-2797(95)03660-1 8620579

[B14] Gan S. Lau E. V. Ng H. K. (2009). Remediation of soils contaminated with polycyclic aromatic hydrocarbons (PAHs). J. Hazard. Mater. 172(2-3), 532–549. 10.1016/j.jhazmat.07,118. 19700241

[B15] Garcia A. C. Araújo B. Birolli W. G. Marques C. Diniz L. Barbosa A. Porto. (2019). Fluoranthene biodegradation by *Serratia sp*. AC-11 immobilized into chitosan beads. Appl. Biochem. Biotech. 188, 1168–1184. 10.1007/s12010-019-02980-9 30854608

[B16] Ghosal D. Ghosh S. Dutta T. K. Ahn Y. (2016). Current state of knowledge in microbial degradation of polycyclic aromatic hydrocarbons (PAHs): a review. Front. Microbiol. 7, 1369. 10.3389/fmicb.2016.01369 27630626 PMC5006600

[B17] Gong B. Wu P. Ruan B. Zhang Y. Lai X. Yu L.. (2018). er al. (2018). Differential regulation of phenanthrene biodegradation process by kaolinite and quartz and the underlying mechanism. J. Hazard. Mater. 349, 51–59. 10.1016/j.jhazmat.01046 29414752

[B18] Gong Y. Z. Niu Q. Y. Liu Y. G. Dong J. Xia M. M. (2022). Development of multifarious carrier materials and impact conditions of immobilised microbial technology for environmental remediation: a review. Environ. Pollut. 314, 120232. 10.1016/j.envpol.2022.120232 36155222

[B19] Gosai H. B. Panseriya H. Z. Patel P. G. Patel A. C. Shankar A. Varjani S.. (2022). Exploring bacterial communities through metagenomics during bioremediation of polycyclic aromatic hydrocarbons from contaminated sediments. Sci. Total. Environ. 842. 10.1016./j.scitotenv.2022.156794 35738384

[B20] Grossman J. N. Kowal S. F. Stubbs A. D. Cawley C. N. Kahan T. F. (2019). Anthracene and pyrene photooxidation kinetics in saltwater environments. Acs. Earth. Space. Chem. 3, 2695–2703. 10.1021/acsearthspacechem.9b00218

[B21] Gu D. Xiang X. Wu Y. Zeng J. Lin X. (2022). Synergy between fungi and bacteria promotes polycyclic aromatic hydrocarbon cometabolism in lignin-amended soil. J. Hazard. Mater. 425, 127958. 10.1016/j.jhazmat.2021.127958 34894508

[B22] Gu H. Yan K. You Q. Chen Y. Pan Y. Wang H.. (2021). Soil indigenous microorganisms weaken the synergy of *Massilia sp*. WF1 and Phanerochaete chrysosporium in phenanthrene biodegradation. Sci. Total Environ. 781, 146655. 10.1016/j.scitotenv.2021.146655 33798893

[B23] Guha S. Peters C. A. Jaffé P. R. (1999). Multisubstrate biodegradation kinetics of naphthalene, phenanthrene, and pyrene mixtures. Biotechnol. Bioeng. 65, 491–499. 10.1002/(SICI)1097-0290(19991205)65:5<491::AID-BIT1>3.0.CO;2-H 10516574

[B24] Guo J. Yang S. He Q. Chen Y. Zheng F. Zhou H.. (2021). Improving benzo (a) pyrene biodegradation in soil with wheat straw-derived biochar amendment: performance, microbial quantity, CO_2_ emission, and soil properties. J. Anal. Appl. Pyrol. 156, 105132. 10.1016/j.jaap.2021.105132

[B25] Haritash A. (2020). A comprehensive review of metabolic and genomic aspects of PAH-degradation. Arch. Microbiol. 202, 2033–2058. 10.1007/s00203-020-01929-5 32506150

[B26] Hathwaik L. T. Thomson J. G. Thilmony R. (2021). Gene assembly in agrobacterium via nucleic acid transfer using recombinase technology (GAANTRY). rice genome engineering and gene editing. Methods Prot. 3, 3–17. 10.1007./978-1-0716-1068-8_1 33471321

[B27] Hunter R. D. Ekunwe S. I. Dodor D. E. Hwang H-M. Ekunwe L. (2005). *Bacillus subtilis* is a potential degrader of pyrene and benzo[a]pyrene. Int. J. Env. Res. Pub. He. 2, 267–271. 10.3390/ijerph2005020010 16705827 PMC3810630

[B28] Imam A. Kanaujia P. K. Ray A. Suman S. K. (2021). Removal of petroleum contaminants through bioremediation with integrated concepts of resource recovery: a review. Indian. J. Microbiol. 61, 250–261. 10.1007/s12088-021-00928-4 34294990 PMC8263831

[B29] Isaac P. Martínez F. L. Bourguignon N. Sánchez L. A. Ferrero M. A. (2015). Improved PAHs removal performance by a defined bacterial consortium of indigenous *Pseudomonas* and actinobacteria from Patagonia, Argentina. Int. Biodeter. Biodegr. 101, 23–31 10.1016/j.ibiod.03014

[B30] Jaiswal S. Shukla P. (2020). Alternative strategies for microbial remediation of pollutants via synthetic biology. Front. Microbiol. 11, 808. 10.3389/fmicb.2020.00808 32508759 PMC7249858

[B31] Jia X. He Y. Jiang D. Liu C. Lu W. (2019). Construction and analysis of an engineered Escherichia coli-*Pseudomonas aeruginosa* co-culture consortium for phenanthrene bioremoval. Biochem. Eng. J. 148, 214–223 10.1016/j.bej.05010

[B32] Jiang X. Mao Z. Zhong L. Yu J. Tang Y. (2022). Strategy to promote the biodegradation of phenanthrene in contaminated soil by a novel bacterial consortium in slurry bioreactors. Int. J. Env. Res. Pub. He. 19, 5515. 10.3390./ijerph19095515 35564911 PMC9101024

[B33] Jiang Y. Zhang Z. Zhang X. (2018). Co-biodegradation of pyrene and other PAHs by the bacterium Acinetobacter johnsonii. Ecotox. Environ. Safe. 163, 465–470. 10.1016/j.ecoenv.07,65 30075449

[B34] Johnston J. E. Lim E. Roh H. (2019). Impact of upstream oil extraction and environmental public health: a review of the evidence. Sci. Total. Environ. 657, 187–199 10.1016/j.scitotenv.11483 30537580 PMC6344296

[B35] Jumper J. Evans R. Pritzel A. Green T. Figurnov M. Ronneberger O.. (2021). Highly accurate protein structure prediction with AlphaFold. Nature 596, 583–589. 10.1038/s41586-021-03819-2 34265844 PMC8371605

[B36] Khan M. J. Rai A. Ahirwar A. Sirotiya V. Mourya M. Mishra S.. (2021). Diatom microalgae as smart nanocontainers for biosensing wastewater pollutants: recent trends and innovations. Bioengineered 12, 9531–9549. 10.1080/21655979.2021.1996748 34709977 PMC8810035

[B37] Khandelwal A. Nain L. Singh S. B. Varghese E. Sharma A. Gupta S.. (2021). Bacteria and fungi mediated degradation of poly aromatic hydrocarbons and effect of surfactant Tween-80. Int. J. Environ. An. Ch. 5, 1–16. 10.1080./03067319.2021.2015584

[B38] Komor A. C. Zhao K. T. Packer M. S. Gaudelli N. M. (2017). Improved base excision repair inhibition and bacteriophage Mu Gam protein yields C: G-to-T: a base editors with higher efficiency and product purity. Sci. Adv. 3, eaao4774. 10.1126./sciadv.aao4774 28875174 PMC5576876

[B39] Kong L. Gao Y. Zhou Q. Zhao X. Sun Z. (2018). Biochar accelerates PAHs biodegradation in petroleum-polluted soil by biostimulation strategy. J. Hazard. Mater. 343, 276–284. 10.1016/j.jhazmat.09040 28988053

[B40] Kong X. Dong R. King T. Chen F. Li H. (2022). Biodegradation potential of *Bacillus sp*. PAH-2 on PAHs for oil-contaminated seawater. Molecules. 27, 687. 10.3390./molecules27030687 35163953 PMC8839208

[B41] Kuppusamy S. Thavamani P. Venkateswarlu K. Lee Y. B. Naidu R. Megharaj M.. (2017). Remediation approaches for polycyclic aromatic hydrocarbons (PAHs) contaminated soils: Technological constraints, emerging trends and future directions. Chemosphere. 168, 944–968. 10.1016/j.chemosphere.10115 27823779

[B42] Lapinaite A. Knott G. J. Palumbo C. M. Lin-Shiao E. Richter M. F. Zhao KT.. (2020). DNA capture by a CRISPR-Cas9–guided adenine base editor. Science 369, 566–571. 10.1126/science.abb1390 32732424 PMC8598131

[B43] Li J. Chen W. Zhou W. Wang Y. Deng M. Zhou S.. (2021a). Synergistic degradation of pyrene by *Pseudomonas aeruginosa* PA06 and *Achromobacter sp*. AC15 with sodium citrate as the co-metabolic carbon source. Ecotoxicology 30, 1487–1498. 10.1007/s10646-020-02268-3 32844301

[B44] Li J. Tang Q. Li Y. Fan Y. Y. Li F. H. Wu JH.. (2020). Rediverting Electron Flux with an Engineered CRISPR-ddAsCpf1 System to Enhance the Pollutant Degradation Capacity of Shewanella oneidensis. Environ. Sci. Technol. 54, 3599–3608. 10.1021/acs.est.9b06378 32062962

[B45] Li M. Yin H. Zhu M. Yu Y. Lu G. Dang Z.. (2021b). Co-metabolic and biochar-promoted biodegradation of mixed PAHs by highly efficient microbial consortium QY1. J. Environ. Sci. 107, 65–76. 10.1016/j.jes.02002 34412788

[B46] Li Y. Li W. Ji L. Song F. Li T. Fu X.. (2021c). Effects of salinity on the biodegradation of polycyclic aromatic hydrocarbons in oilfield soils emphasizing degradation genes and soil enzymes. Front. Microbiol. 12, 824319. 10.3389/fmicb.2021.824319 35087508 PMC8787140

[B47] Liang C. Huang Y. Wang H. Parales R. E. (2019). *pahE*, a functional marker gene for polycyclic aromatic hydrocarbon-degrading bacteria. Appl. Environ. Microb. 85, 18. 10.1128./aem.02399-18 30478232 PMC6344622

[B48] Liang C. Ye Q. Huang Y. Zhang Z. Wang C. Wang Y.. (2023). Distribution of the new functional marker gene (*pahE*) of aerobic polycyclic aromatic hydrocarbon (PAHs) degrading bacteria in different ecosystems. Sci. Total. Environ. 865, 161233. 10.1016/j.scitotenv.2022.161233 36586685

[B49] Liu X. Ding W. Jiang H. (2017). Engineering microbial cell factories for the production of plant natural products: from design principles to industrial-scale production. Microb. Cell. Fact. 16, 125. 10.1186/s12934-017-0732-7 28724386 PMC5518134

[B50] Llasera Garcia M. Garcia de Llasera MP. (2021). A review on the enzymes and metabolites identified by mass spectrometry from bacteria and microalgae involved in the degradation of high molecular weight PAHs. Sci. Total. Environ. 797, 149035. 10.1016/j.scitotenv.2021.149035 34303250

[B51] Ma Z. Liu J. Dick R. P. Li H. Shen D. Gao Y.. (2018). Rhamnolipid influences biosorption and biodegradation of phenanthrene by phenanthrene-degrading strain *Pseudomonas sp*. Ph6. Environ. Pollut. 240, 359–367. 10.1016/j.envpol.04125 29751332

[B52] Mardani G. Mahvi A. H. Hashemzadeh-Chaleshtori M. Naseri S. Dehghani M. H. Ghasemi-Dehkordi P.. (2017). Application of genetically engineered dioxygenase producing pseudomonas putida on decomposition of oil from spiked soil. Jundishapur. J. Nat. Ph. 12, e64313. 10.5812/jjnpp.64313

[B53] Martinez-Toledo A. del Carmen Cuevas-Diaz M. Guzman-Lopez O. López-Luna J. Ilizaliturri-Hernandez C. (2022). Evaluation of in situ biosurfactant production by inoculum of *P*. putida and nutrient addition for the removal of polycyclic aromatic hydrocarbons from aged oil-polluted soil. Biodegradation 33, 135–155. 10.1007/s10532-022-09973-2 35092539

[B54] Maxel S. King E. Zhang Y. Luo R. Li H. (2020). Leveraging oxidative stress to regulate redox balance-based, in vivo growth selections for oxygenase engineering. Acs. Synth. Biol. 9, 3124–3133. 10.1021/acssynbio.0c00380 32966747 PMC10441625

[B55] Mazarji M. Minkina T. Sushkova S. Mandzhieva S. Bidhendi G. N. Barakhov A.. (2021). Effect of nanomaterials on remediation of polycyclic aromatic hydrocarbons-contaminated soils: a review. J. Environ. Manage. 284, 112023. 10.1016/j.jenvman.2021.112023 33540196

[B56] Mehetre G. T. Dastager S. G. Dharne M. S. (2019). Biodegradation of mixed polycyclic aromatic hydrocarbons by pure and mixed cultures of biosurfactant producing thermophilic and thermo-tolerant bacteria. Sci. Total. Environ. 679, 52–60 10.1016/j.scitotenv.04376 31082602

[B57] Meng L. Li W. Bao M. Sun P. (2019). Effect of surfactants on the solubilization, sorption and biodegradation of benzo (a) pyrene by *Pseudomonas aeruginosa* BT-1. J. Taiwan. *Inst. Chem*. E. 96, 121–130. 10.1016/j.jtice.01007

[B58] Mille-Lindblom C. Fischer HJ. Tranvik L. (2006). Antagonism between bacteria and fungi: substrate competition and a possible tradeoff between fungal growth and tolerance towards bacteria. Oikos. 113, 233–242. 10.1111/j.2006.0030-1299.14337.x

[B59] Moscoso F. Teijiz I. Deive F. J. Sanroman M. A. (2012). Efficient PAHs biodegradation by a bacterial consortium at flask and bioreactor scale. Bioresour. Technol. 119, 270–6. 10.1016/j.biortech.05095 22738812

[B60] Omoni V. T. Ibeto C. N. Lag-Brotons A. J. Bankole P. O. Semple K. T. (2022). Impact of lignocellulosic waste-immobilised white-rot fungi on enhancing the development of (14)C-phenanthrene catabolism in soil. Sci. Total. Environ. 811, 152243. 10.1016/j.scitotenv.2021.152243 34921880

[B61] Pant G. Garlapati D. Agrawal U. Prasuna R. G. Mathimani T. Pugazhendhi A.. (2021). Biological approaches practised using genetically engineered microbes for a sustainable environment: a review. J. Hazard. Mater. 405, 124631. 10.1016/j.jhazmat.2020.124631 33278727

[B62] Patel A. B. Jain K. R. Manvar T. Desai C. Madamwar D. (2022). Enriched bacterial community efficiently degrade polycyclic aromatic hydrocarbons in soil ecosystem: Insights from a mesocosms study. Biochem. Eng. J. 185. 10.1016./j.bej.2022.108516

[B63] Patel A. B. Mahala K. Jain K. Madamwar D. (2018). Development of mixed bacterial cultures DAK11 capable for degrading mixture of polycyclic aromatic hydrocarbons (PAHs). Bioresour. Technol. 253, 288–296 10.1016/j.biortech.01049 29353758

[B64] Patel A. B. Singh S. Patel A. Jain K. Amin S. Madamwar D.. (2019). Synergistic biodegradation of phenanthrene and fluoranthene by mixed bacterial cultures. Bioresour. Technol. 284, 115–120. 10.1016/j.biortech.03097 30927648

[B65] Premnath N. Mohanrasu K. Guru Raj Rao R. Dinesh GH. Prakash GS.. (2021). A crucial review on polycyclic aromatic Hydrocarbons—Environmental occurrence and strategies for microbial degradation. Chemosphere. 280, 608. 10.1016./j.chemosphere.2021.130608 33962296

[B66] Qin R. Xu T. Jia X. (2022). Engineering *Pseudomonas putida* to produce rhamnolipid biosurfactants for promoting phenanthrene biodegradation by a two-species microbial consortium. Microbiol. Spectr. 10, e00910–22. 10.1128/spectrum.00910-22 35730952 PMC9431653

[B67] Rafeeq H. Afsheen N. Rafique S. Arshad A. Intisar M. Hussain A.. (2023). Genetically engineered microorganisms for environmental remediation. Chemosphere. 310, 136751. 10.1016/j.chemosphere.2022.136751 36209847

[B68] Sharma P. Bano A. Singh S. P. Sharma S. Xia C. Nadda A. K.. (2022). Engineered microbes as effective tools for the remediation of polyaromatic aromatic hydrocarbons and heavy metals. Chemosphere. 306, 135538. 10.1016/j.chemosphere.2022.135538 35792210

[B69] Sivaram A. K. Logeshwaran P. Lockington R. Naidu R. Megharaj M. (2019). (2019). Low molecular weight organic acids enhance the high molecular weight polycyclic aromatic hydrocarbons degradation by bacteria. Chemosphere. 222, 132–140 10.1016/j.chemosphere.0110 30703652

[B70] Srivastava S. Kumar M. (2019). Biodegradation of polycyclic aromatic hydrocarbons (PAHs): a sustainable approach. Sust. Green Technol. Environ. Manag. 3, 111–139. 10.1007./978-981-13-2772-8_636216116

[B71] Su Y. Sun S. Liu Q. Zhao C. Li L. Chen S.. (2022). Characterization of the simultaneous degradation of pyrene and removal of Cr (VI) by a bacteria consortium YH. Sci. Total. Environ. 853, 158388. 10.1016/j.scitotenv.2022.158388 36049693

[B72] Sun S. Wang H. Yan K. Lou J. Ding J. Snyder S. A.. (2021). Metabolic interactions in a bacterial co-culture accelerate phenanthrene degradation. J. Hazard. Mater. 403, 123825. 10.1016/j.jhazmat.2020.123825 33264917

[B73] Sun Y. Liu L. Zeng J. Wu Y. Lin X. (2020). Enhanced cometabolism of benzo(a)anthracene by the lignin monomer vanillate is related to structural and functional responses of the soil microbiome. Soil. Biol. Biochem. 149, 7098. 10.1016./j.soilbio.2020.107908

[B74] Varjani S. Pandey A. Upasani V. N. (2020). Oilfield waste treatment using novel hydrocarbon utilizing bacterial consortium — A microcosm approach. Sci. Total. Environ. 745. 10.1016./j.scitotenv.2020.141043 32717605

[B75] Varjani S. Upasani V. N. (2021). Bioaugmentation of *Pseudomonas aeruginosa* NCIM 5514 - A novel oily waste degrader for treatment of petroleum hydrocarbons. Bioresour. Technol. 319, 124240. 10.1016/j.biortech.2020.124240 33254463

[B76] Varjani S. J. (2017). Microbial degradation of petroleum hydrocarbons. Bioresour. Technol. 223, 277–286 10.1016/j.biortech.10037 27789112

[B77] Wang D. Qin L. Liu E. Chai G. Su Z. Shan J.. (2022). Biodegradation performance and diversity of enriched bacterial consortia capable of degrading high-molecular-weight polycyclic aromatic hydrocarbons. Environ. Technol. 43, 4200–4211. 10.1080/09593330.2021.1946163 34148513

[B78] Wu X. Rensing C. Han D. Xiao K-. Q. Dai Y. Tang Z.. (2022). Genome-resolved metagenomics reveals distinct phosphorus acquisition strategies between soil microbiomes. Msystems. 7, e01107–21. 10.1128/msystems.01107-21 35014868 PMC8751388

[B79] Xiang L. Li G. Wen L. Su C. Liu Y. Tang H.. (2021). Biodegradation of aromatic pollutants meets synthetic biology. Syn. Syst. Biotechno. 6, 153–162. 10.1016/j.synbio.06001 34278013 PMC8260767

[B80] Xiao K. Wang R. Liu C. Wang M. Dong W. Pan T.. (2021). Influence of Triton X-100 and β-cyclodextrin on the bioavailability and biodegradation of crystalline phenanthrene covered with biofilms. Process. Biochem. 4, 14. 10.1016/j.procbio.0101425776733

[B81] Xie Y. Yu F. Wang Q. Gu X. Chen W. (2014). Cloning of catechol 2,3-dioxygenase gene and construction of a stable genetically engineered strain for degrading crude oil. Indian. J. Microbiol. 54, 59–64. 10.1007/s12088-013-0411-2 24426168 PMC3889840

[B82] Xu X. Zhou H. Chen X. Wang B. Jin Z. Ji F.. (2019). Biodegradation potential of polycyclic aromatic hydrocarbons by immobilized *Klebsiella sp*. in soil washing effluent. Chemosphere. 223, 140–147. 10.1016/j.chemosphere.01,196. 30772593

[B83] Xu Y. Zhou N-. Y. (2016). Microbial remediation of aromatics-contaminated soil. Front. Env.Sci. Eng. 11, 894. 10.1007./s11783-017-0894-x

[B84] Yu T. Liu X. Ai J. Wang J. Guo Y. Liu X.. (2022). Microbial community succession during crude oil-degrading bacterial enrichment cultivation and construction of a degrading consortium. Front. Microbiol. 13, 1044448. 10.3389/fmicb.2022.1044448 36406435 PMC9672818

[B85] Zhang G. Yang X. Zhao Z. Xu T. Jia X. (2021a). Artificial consortium of three *E. coli* BL21 strains with synergistic functional modules for complete phenanthrene degradation. Acs. Synth. Biol. 11, 162–175. 10.1021/acssynbio.1c00349 34914358

[B86] Zhang L. Li X. Zuo W. Li S. Sun G. Wang W.. (2021b). Root exuded low-molecular-weight organic acids affected the phenanthrene degrader differently: a multi-omics study. J. Hazard. Mater. 414, 125367. 10.1016/j.jhazmat.2021.125367 33677320

[B87] Zhang L. Qiao J. Cui H. Wang M. Li X. (2021c). Using low molecular weight organic acids to enhance microbial degradation of polycyclic aromatic hydrocarbons: current understanding and future perspectives. Water. 13, 446. 10.3390./w13040446

[B88] Zhang L. Qiu X. Huang L. Xu J. Wang W. Li Z.. (2021d). Microbial degradation of multiple PAHs by a microbial consortium and its application on contaminated wastewater. J. Hazard. Mater. 419, 126524. 10.1016/j.jhazmat.2021.126524 34323721

[B89] Zhang L. Yang B. Qu C. Chen G. Qi F. Yu T.. (2022). Construction and degradation performance study of polycyclic aromatic hydrocarbons (PAHs) degrading bacterium consortium. Appl. Sci. 12, 2345. 10.3390./app12052354

[B90] Zhao X. Miao R. Guo M. Shang X. Zhou Y. Zhu J.. (2022). Biochar enhanced polycyclic aromatic hydrocarbons degradation in soil planted with ryegrass: bacterial community and degradation gene expression mechanisms. Sci. Total. Environ. 838(Pt 2), 156076. 10.1016./j.scitotenv.2022.1560 35597344

